# Conservation of the OmpC Porin Among Typhoidal and Non-Typhoidal *Salmonella* Serovars

**DOI:** 10.3389/fimmu.2019.02966

**Published:** 2020-01-09

**Authors:** Nuriban Valero-Pacheco, Joshua Blight, Gustavo Aldapa-Vega, Phillip Kemlo, Marisol Pérez-Toledo, Isabel Wong-Baeza, Ayako Kurioka, Christian Perez-Shibayama, Cristina Gil-Cruz, Luvia E. Sánchez-Torres, Rodolfo Pastelin-Palacios, Armando Isibasi, Arturo Reyes-Sandoval, Paul Klenerman, Constantino López-Macías

**Affiliations:** ^1^Unidad de Investigación Médica en Inmunoquímica, Hospital de Especialidades del Centro Médico Nacional Siglo XXI, Instituto Mexicano del Seguro Social, Mexico City, Mexico; ^2^Departamento de Inmunología, Escuela Nacional de Ciencias Biológicas, Instituto Politécnico Nacional, Mexico City, Mexico; ^3^Nuffield Department of Medicine, The Henry Wellcome Building for Molecular Physiology, The Jenner Institute, University of Oxford, Oxford, United Kingdom; ^4^Peter Medawar Building for Pathogen Research, University of Oxford, Oxford, United Kingdom; ^5^Institute of Immunobiology, Kantonsspital St. Gallen, St. Gallen, Switzerland; ^6^Facultad de Química, Universidad Nacional Autónoma de México, Mexico City, Mexico; ^7^Visiting Professor of Immunology, Nuffield Department of Medicine, University of Oxford, Oxford, United Kingdom; ^8^Mexican Translational Immunology Research Group, FOCIS Centres of Excellence, Cuernavaca, Mexico

**Keywords:** *Salmonella*, OmpC, immunogenicity, non-typhoidal, porin, vaccine, typhoid, salmonellosis

## Abstract

*Salmonella enterica* infections remain a challenging health issue, causing significant morbidity and mortality worldwide. Current vaccines against typhoid fever display moderate efficacy whilst no licensed vaccines are available for paratyphoid fever or invasive non-typhoidal salmonellosis. Therefore, there is an urgent need to develop high efficacy broad-spectrum vaccines that can protect against typhoidal and non-typhoidal *Salmonella*. The *Salmonella* outer membrane porins OmpC and OmpF, have been shown to be highly immunogenic antigens, efficiently eliciting protective antibody, and cellular immunity. Furthermore, enterobacterial porins, particularly the OmpC, have a high degree of homology in terms of sequence and structure, thus making them a suitable vaccine candidate. However, the degree of the amino acid conservation of OmpC among typhoidal and non-typhoidal *Salmonella* serovars is currently unknown. Here we used a bioinformatical analysis to classify the typhoidal and non-typhoidal *Salmonella* OmpC amino acid sequences into different clades independently of their serological classification. Further, our analysis determined that the porin OmpC contains various amino acid sequences that are highly conserved among both typhoidal and non-typhoidal *Salmonella* serovars. Critically, some of these highly conserved sequences were located in the transmembrane β-sheet within the porin β-barrel and have immunogenic potential for binding to MHC-II molecules, making them suitable candidates for a broad-spectrum *Salmonella* vaccine. Collectively, these findings suggest that these highly conserved sequences may be used for the rational design of an effective broad-spectrum vaccine against *Salmonella*.

## Introduction

*Salmonella enterica* infections remain a significant worldwide health problem, accounting for more than 120 million cases and approximately 1 million deaths annually (1, 2). These high morbidity and mortality rates are caused mainly by enteric fevers (typhoid and paratyphoid) and by non-typhoidal *Salmonella* (NTS) gastroenteritis (1–3). Furthermore, invasive NTS bacteremia (iNTS) is a common complication observed in immunocompromised adults and in young children with severe malaria and malnutrition (4). The current available licensed vaccines against *Salmonella* are the oral live attenuated Ty21a, the Vi capsular polysaccharide (Vi CPS), and the Vi-tetanus toxoid conjugate (Vi-TT), which only target the Typhi serovar, and have shown variable efficacy; 50% (95% CI 35–61%) for Ty21a, 55% (95% CI 30–70%) for Vi-CPS, and 54.6% (95% CI 26.8–71.8%) for Vi-TT (5, 6), while no licensed vaccines against iNTS are currently available (7). Although cross-reactivity through vaccination with the Ty21a vaccine can be induced against Paratyphi A, B, and Enteritidis serovars (8, 9), cross-protection has been reported only against Paratyphi B (10). Therefore, there is an urgent need for the development of novel broad-spectrum vaccines against *Salmonella*, which must be based on shared key structural components that induce protective immune responses against typhoidal and NTS serovars.

Porins are one of the most abundant outer-membrane proteins (Omp) in Gram-negative bacteria, which play a crucial role in the diffusion of small hydrophilic compounds, and are essential for bacterial survival and pathogenicity (11, 12). Porins are β-barrel structures consisting of 16 β-sheets (β), with 8 internal periplasmic turns (T) and 8 extracellular loops (L) (13, 14). *Salmonella* and other Gram-negative bacteria express two major porins, OmpC and OmpF (15–17). We have previously shown that *S*. Typhi OmpC and OmpF porins efficiently elicit innate immune responses through the TLR-mediated activation of antigen-presenting cells (18), which induce long-lasting porin-specific bactericidal antibody and cell-mediated immune responses (19–22). However, the basis of antigen specificity of *Salmonella* porins is not well-understood. Previous studies have shown that the porin OmpC shows a high degree of homology in terms of sequence and structure among Enterobacteriaceae porins (11, 13, 15, 23, 24). Therefore, antibody and cell-mediated cross-reactivity among *Salmonella* serovar porins has been widely reported in mouse models (19, 24–28). However, the degree of amino acid conservation of the porin OmpC among typhoidal and NTS serovars remains unknown. Through bioinformatics, we found that the typhoidal and NTS OmpC amino acid sequences can be classified into eight different clades that are independent of serovar classification. In addition, we found that the porin OmpC contains three distinct amino acid sequences, which are highly conserved among typhoidal and NTS serovars. These highly conserved sequences are located along the transmembrane β-sheet domains within the porin β-barrel. Furthermore, we found that one of the highly conserved OmpC sequences is present exclusively in *Salmonella* and not in other Enterobacterial porins and has the potential of binding to MHC-II molecules. Collectively, our results show that the porin OmpC of *Salmonella* contains highly conserved amino acid sequences, which could be used for the rational design of an effective, broad-spectrum vaccine against *Salmonella*.

## Materials and Methods

### Conservation Analysis

Full-length protein sequences for OmpC porin from typhoidal and NTS serovars (Typhi, Paratyphi A, B, C, Typhimurium, Enteritidis, Dublin, and Gallinarum) were collected from the NCBI Entrez protein database using Taxonomy IDs (Txid). Subsequently, sequences for OmpC from all serovars were each aligned using Clustal Omega standalone binary 1.2.1 (29) and used to create a neighbor-joining tree using the Jukes-Cantor model resampled with 100 bootstraps and samples separated into clades (8 clades). Porin conservation was assessed using in-house developed software, based on a sliding window approach. Amino acid conservation within each clade was assessed using a 15 amino acid window with a mean conservation value (between 0 and 1) given for each window, determined by amino acid similarity. Windows with a mean value less than the first quartile of all windows was classed as conserved (intra-clade). Zero (0) represents a fully conserved window. This was used to generate an intra-conservation plot representing the mean window conservation across the entire proteome. Subsequently, conservation across clades (inter-clade) was assessed by identifying windows at the same position across clades that were conserved within their respective clades (i.e., mean window value below the first quartile) and given an arbitrary value between 0 and 1000 to indicate the magnitude of inter-clade conservation given. A consensus was created from the clades with shared conservation.

### Porin Visualization

*Salmonella* Typhi OmpC full-length amino acid sequence was obtained from UniProt (P0A264-OMPC SALTI) (30), and the secondary structure was visualized using PDBSum (31). The three-dimensional *S*. Typhi OmpC (32) porin structure was obtained from PDB (ID: 3UU2). Geneious version 8.1.7, created by Biomatters (33), was used for porin 3D visualization.

### MHC-II Peptide-Binding Prediction

To evaluate the potential immunogenicity of the OmpC conserved sequences, the Immuno Epitope Database (IEDB, https://www.iedb.org) MHC-II binding prediction tool was used. The MHCII binding predictions were performed on Oct/18/2019 using the IEDB analysis resource Consensus tool (34, 35). The predicted output is given in units of IC_50_nM for combinatorial library and SMM_align; hence, a lower number indicates a higher affinity. According to the IEDB, as a rough guideline, peptides with IC_50_ values <50 nM are considered high affinity, <500 nM intermediate affinity and <5,000 nM low affinity. Most known epitopes have high or intermediate affinity (36).

### BLAST Analysis

The conserved sequences of *Salmonella* OmpC porin were compared against Non-Redundant (nr) GenBank database using the standard protein Basic Local Alignment Search Tool (BLAST), BLASTP 2.10.0+, using the default options (37). Fast minimum evolution pairwise alignment trees were constructed using the default options of BLASTP 2.10.0+ (max seq. difference 0.85, Grishin distance).

### Statistical Analysis

Statistics were calculated using linear regression in GraphPad Prism 6.0. *P* values ≤ 0.05 were considered as significant.

## Results

### Identification of Conserved Amino Acid Sequences in the Porin OmpC Among Typhoidal and Non-Typhoidal *Salmonella* Serovars

**Figure 1:**
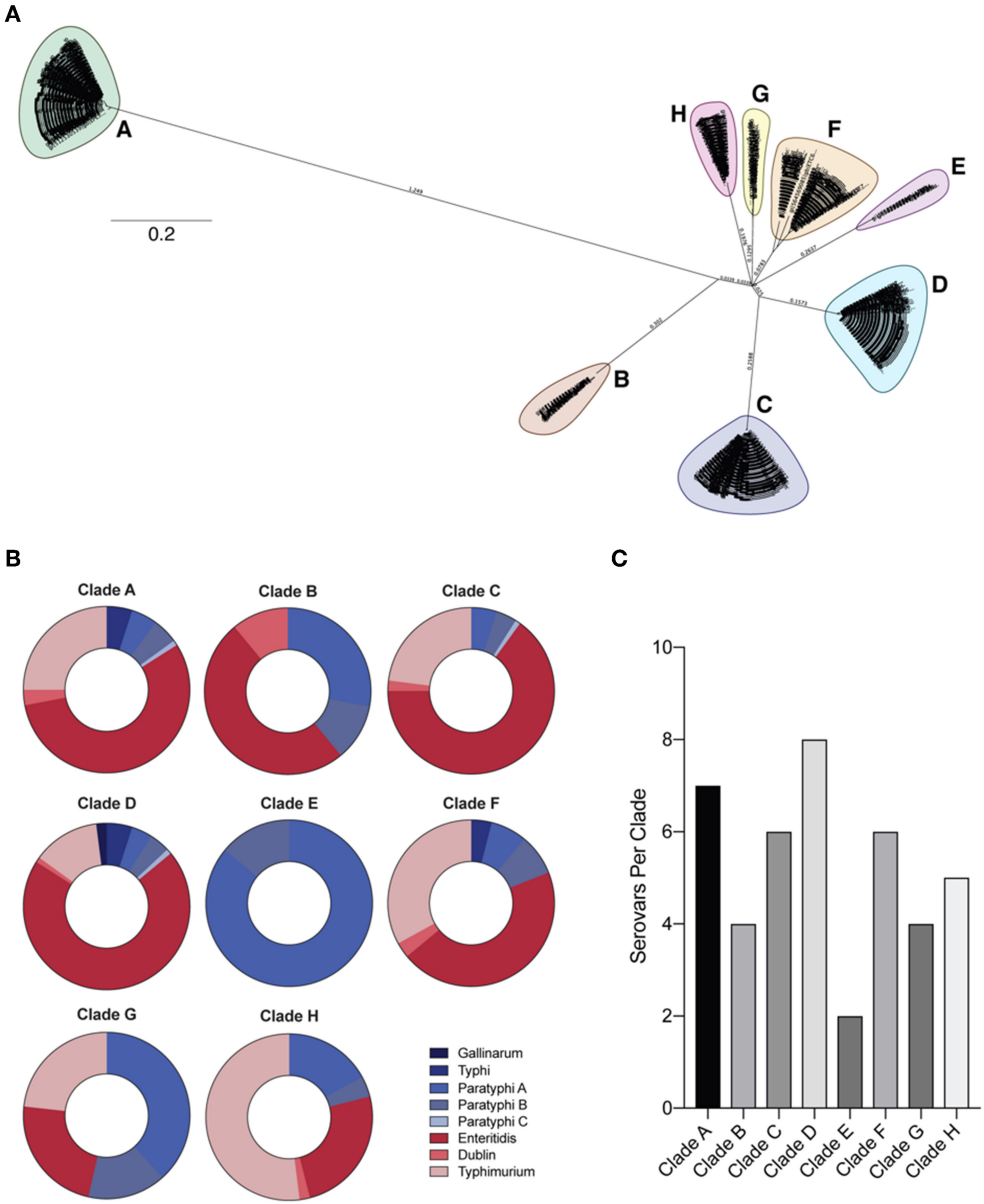
Phylogeny of *Salmonella* OmpC protein. **(A)** Neighbor-joining tree of full-length *Salmonella* OmpC protein sequences. Full-length *Salmonella* OmpC protein sequences used to create a neighbor-joining tree using the Jukes-Cantor model with 100 bootstraps. Outgroups separated into 8 clades (A–H). **(B)** Prevalence of typhoidal and non-typhoidal *Salmonella* serovars among each OmpC clade. Full-length OmpC protein sequences were retrieved from NCBI and aligned using Clustal Omega, and intra-serovar OmpC conservation was assessed using in-house developed software utilizing a sliding window approach (for a detailed description see methods). The percentage of each *Salmonella* serovar OmpC amino acid sequence within *Salmonella* clades is shown. Typhoidal serovars are shown in blue while non-typhoidal serovars are shown in red. **(C)** Number of serovars per clade represented by a bar chart.

To determine the degree of conservation among OmpC amino acid sequences from clinically relevant typhoidal (Typhi, Paratyphi A, B, and C) and non-typhoidal (Typhimurium, Enteritidis, Dublin, and Gallinarum) *Salmonella* serovars (38), we retrieved and aligned 761 *Salmonella* serovar OmpC amino acid sequences and assessed conservation within serovars using in-house developed software (see methods) (Table 1). However, sequences within serovars showed very poor identity, likely due to the serological classification of serovars (39). Therefore, OmpC sequences from these serovars were used to create a neighbor-joining tree and outgroups were classed into 8 separate clades (Figure 1A, Table 2). Each clade contained a mixture of serovars (Figures 1B,C). However, clades A, B, C, D, F, and H consisted of a majority of non-typhoidal serovars, while clades E and G were comprised of mostly typhoidal serovars, with clade E containing only sequences from Paratyphi A and B. As would be expected, the greater the number of sequences per clade the greater the number of serovars it contained (*R*^2^ = 0.7709, ^**^p = 0.00413). We found that Clade D contained sequences from all 8 serovars analyzed, followed by clade A with 7 serovars, C and F with 6 serovars, clade H with 5 serovars, clades B and G with 4 serovars, and clade E with 2 serovars. In addition, we found that the most widely distributed serovars among the clades were Paratyphi A and B, which had sequences present in all of the clades. *Salmonella* Enteritidis was found in all but one of the clades analyzed (clade E), while Typhimurium and Dublin were found in 6 clades. Typhi and Paratyphi C serovars were only present in 3 clades, whereas Gallinarum was only found in one clade.

**Table 1:** Number of full-length amino acid sequences retrieved from the NCBI Entrez Protein database for the selected *Salmonella* OmpC porin.

**Serovar**	**Txid**	**Number of sequences**
Typhi	90370	24
Paratyphi A	54388	57
Paratyphi B	57045	40
Paratyphi C	57046	6
Dublin	98360	19
Enteritidis	149539	426
Typhimurium	90371	186
Gallinarum	594	3
	Total	761

**Table 2:** Number of full-length amino acid sequences for each clade of *Salmonella* OmpC porin sequences.

**Clade**	**Number of sequences**
A	208
B	18
C	181
D	169
E	7
F	117
G	13
H	48
Total	761

**Figure 2:**
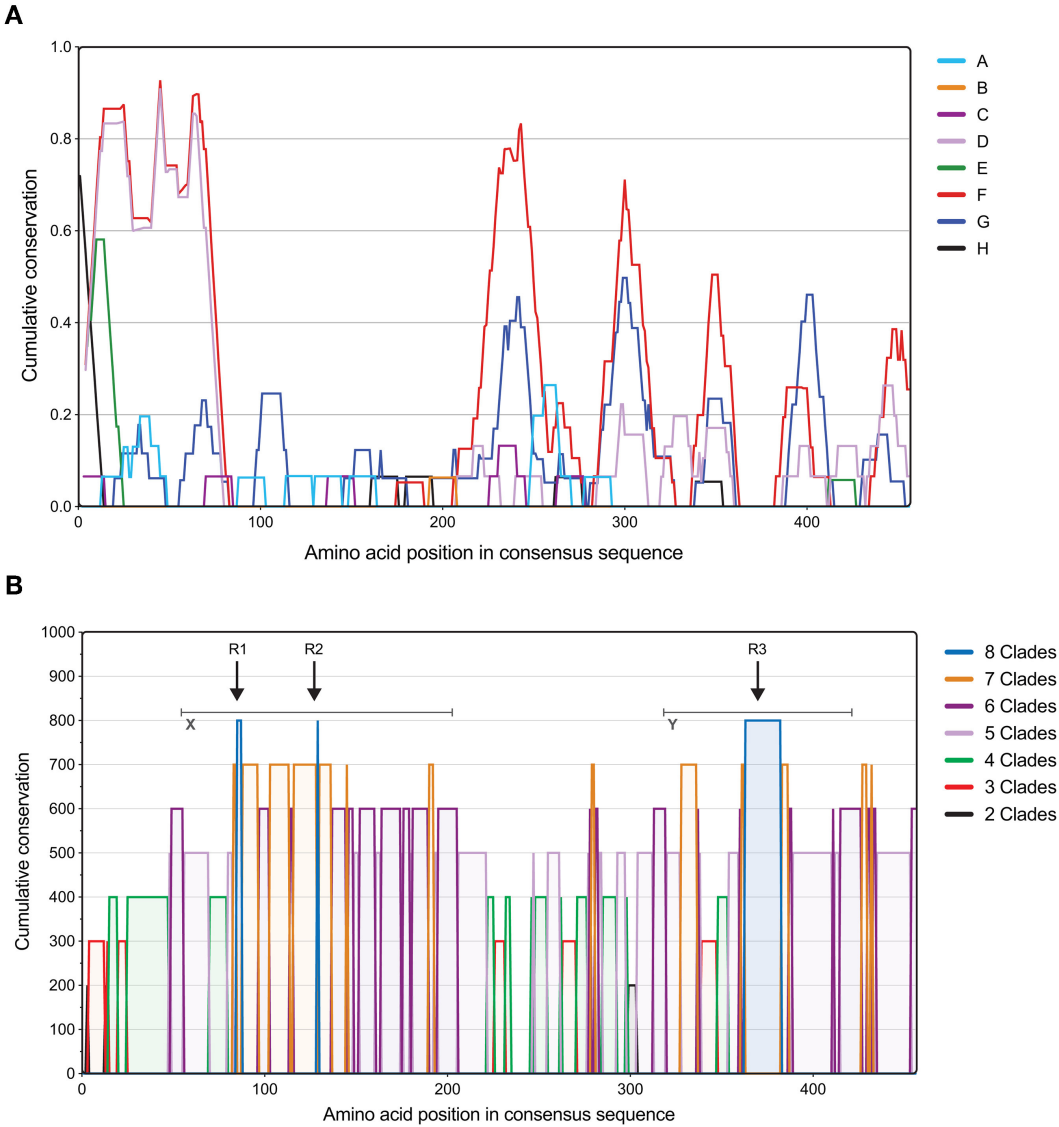
Assessment of *Salmonella* OmpC clade conservation. **(A)** Intra-clade conservation of OmpC porin within typhoidal and non-typhoidal *Salmonella* serovars. Conservation of each *Salmonella* clade of the OmpC protein identified using a 15 amino acid sliding window approach (in-house software; for a detailed description see methods). The measure of OmpC amino acid conservation within *Salmonella* clades is shown in Y-axis (0–1), while X-axis shows the position in the aligned amino acid consensus sequence. A conservation value below the first quartile was classed as conserved for each clade. **(B)** Inter-clade conservation patterns in the protein sequence of OmpC porin among typhoidal and non-typhoidal *Salmonella* serovars. Full-length OmpC protein sequences were retrieved from NCBI and aligned using Clustal Omega, and inter-serovar conservation was assessed using in-house developed software. The measure of OmpC amino acid conservation between *Salmonella* clades is shown in Y-axis, while X-axis shows the position in the aligned amino acid consensus sequence. Colors indicate the number of clades that share conservation between each other. Arrows indicate the regions conserved among all *Salmonella* clades and gray bars indicate regions of distinct cross-clade conservation (see Table 1).

Next, we assessed the degree of conservation of full-length OmpC sequences within each clade (intra-clade; Figure 2A). Analysis showed a pattern of diversity and conservation across the protein unique to each clade, however some clades showed similar conservation fingerprints. For example, clades D and F showed a similar trend at the N-terminus, whereas clades F and G were more similar toward the C-terminus. There was no significant correlation between either the number of serovars per clade and the median conservation value for that clade (*R*^2^ = 0.09655, *p* = 0.6412) and the number of sequences per clade and the median conservation value for that clade (*R*^2^ = 0.008383, *p* = 0.8293).

Subsequently, conservation between clades was assessed (Figure 2B), which identified 5, 15, 23, 28, 16, 8, and 2% of the protein covered by regions conserved in all 8 or 7, 6, 5, 4, 3, and 2 clades respectively. Whereas, 3% of the protein sequence showed no conservation across clades, located centrally at position 236–246. Remarkably, when comparing more clades (>5) two regions of distinct cross clade conservation could be seen located at the 50–200 (X) and 320–430 (Y) amino acid positions, suggesting two regions of functional importance. Within these locations there were three regions (R1–R3) that showed a high degree of conservation among all of the *Salmonella* clades analyzed (Table 3). Collectively, these data show that the OmpC porin contains distinct amino acid sequences that are highly conserved among typhoidal and non-typhoidal *Salmonella* serovars.

**Table 3:** Conserved regions in the amino acid consensus sequences for the porin OmpC among the *Salmonella* serovars analyzed.

**Conserved region**	**Amino acid sequence**
R1	KGETQINDQLTGY
R2	WTRLAFAGLKFA
R3	GFANKTQNFEVVAQYQFDFGLRPSQAYLSKG

### The Conserved Amino Acid Sequences Are Located Along the β-Sheets of OmpC Porin

**Figure 3:**
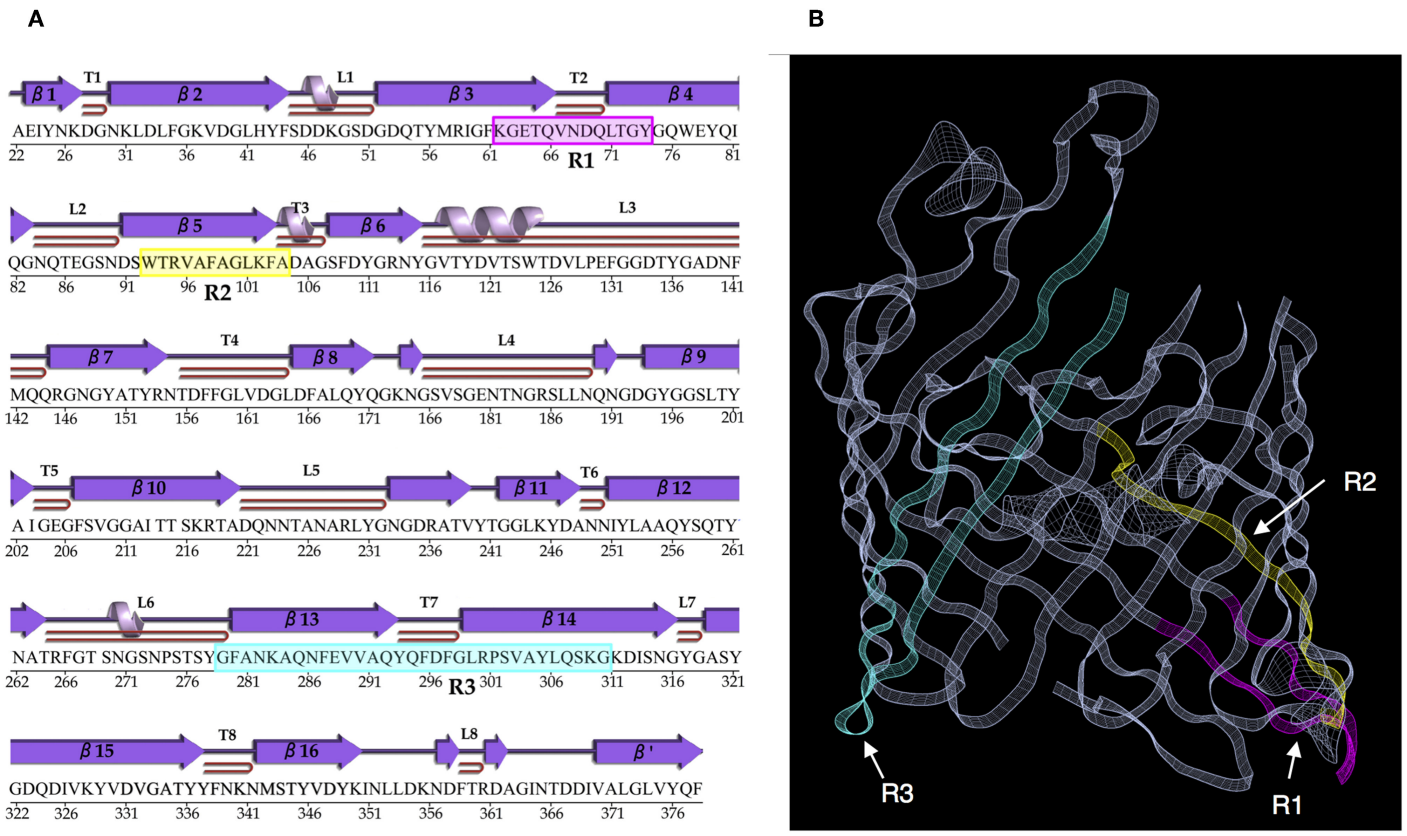
The amino acid sequences conserved among *Salmonella* OmpC porin clades are located along the β-sheets of the β-barrel. **(A)** The secondary structure of the *S*. Typhi OmpC porin was modeled with PDBsum (ID: 3UU2). α-helices and β-sheets are numbered and shown in purple. The periplasmic turns are labeled T1–T8, and the extracellular loops are labeled L1–L8. The porin amino acid sequence is shown without the signal peptide. **(B)** Crystal structure of a *S*. Typhi OmpC monomer. **(A,B)** The conserved regions (R1–R3) among *Salmonella* clades are highlighted in colors, R1 (magenta), R2 (yellow), and R3 (cyan).

Next, we sought to identify the location of the conserved regions along the secondary structure of *S*. Typhi OmpC, the only available crystal structure of a *Salmonella* OmpC porin reported to date (Figure 3A) (32). Our results show that the sequence of the conserved region R1 (KGETQINDQLTGY) was located partially along the β3 β-sheet, the periplasmic turn T3, and part of the β4 β-sheet. The conserved region R2 (WTRLAFAGLKFA) was located along the β5 β-sheet. Finally, the conserved region R3 (GFANKTQNFEVVAQYQFDFGLRPSQAYLSKG) was located along the β13 β-sheet, the periplasmic turn T7, and the β14 β-sheet. The visualization the conserved amino acid sequences on the crystal *S*. Typhi OmpC porin structure showed that most of the conserved sequences were distributed along the porin β-barrel (Figure 3B). Collectively, our data show that the amino acid sequences conserved among *Salmonella* clades are located along the β-sheets and periplasmic turns of the OmpC porin β-barrel.

### The Conserved Amino Acid Sequence R1 Is Exclusive for *Salmonella*

**Figure 4:**
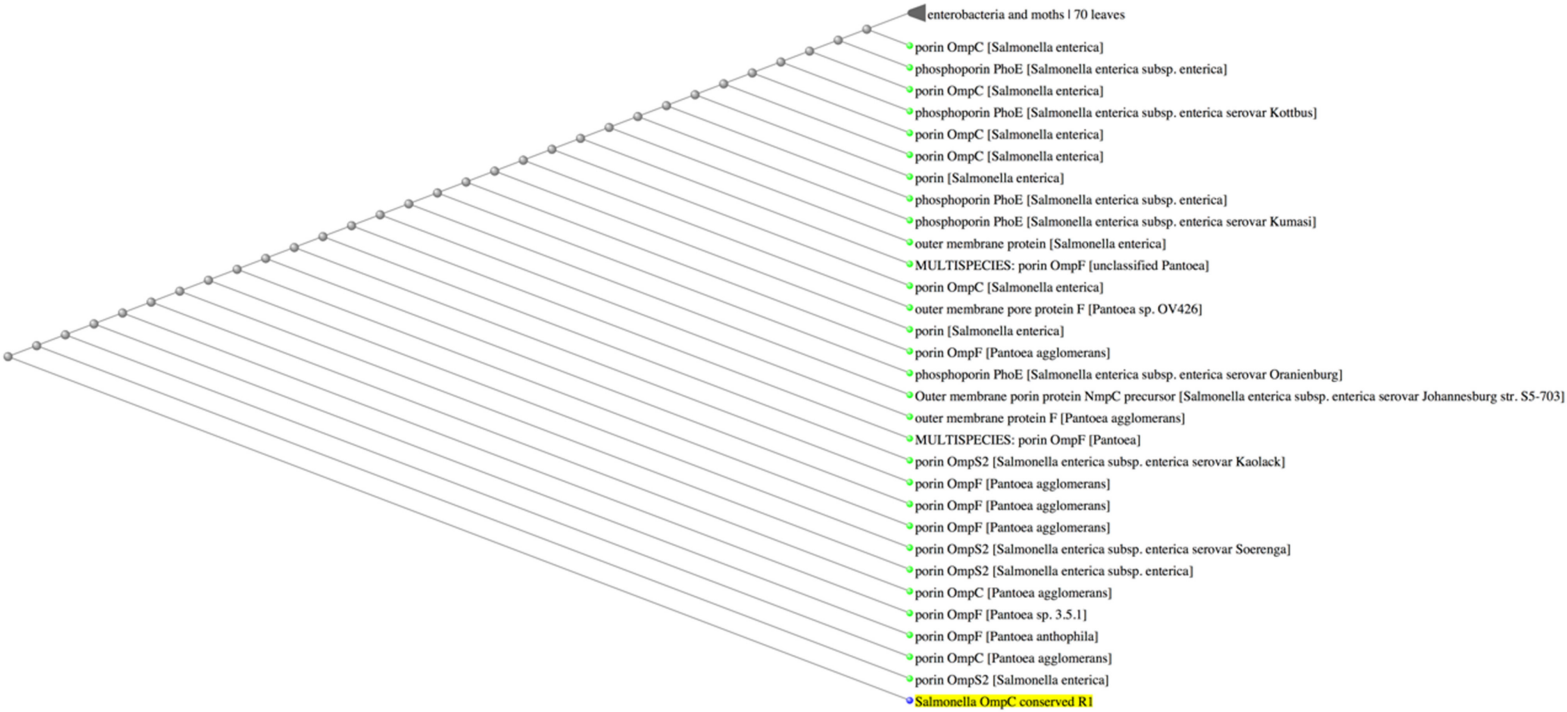
The amino acid sequence of R1 is exclusive of *Salmonella* porins. Fast minimum evolution tree between the R1 sequence and the Non-Redundant (nr) GenBank database calculated by BLASTp.

**Figure 5:**
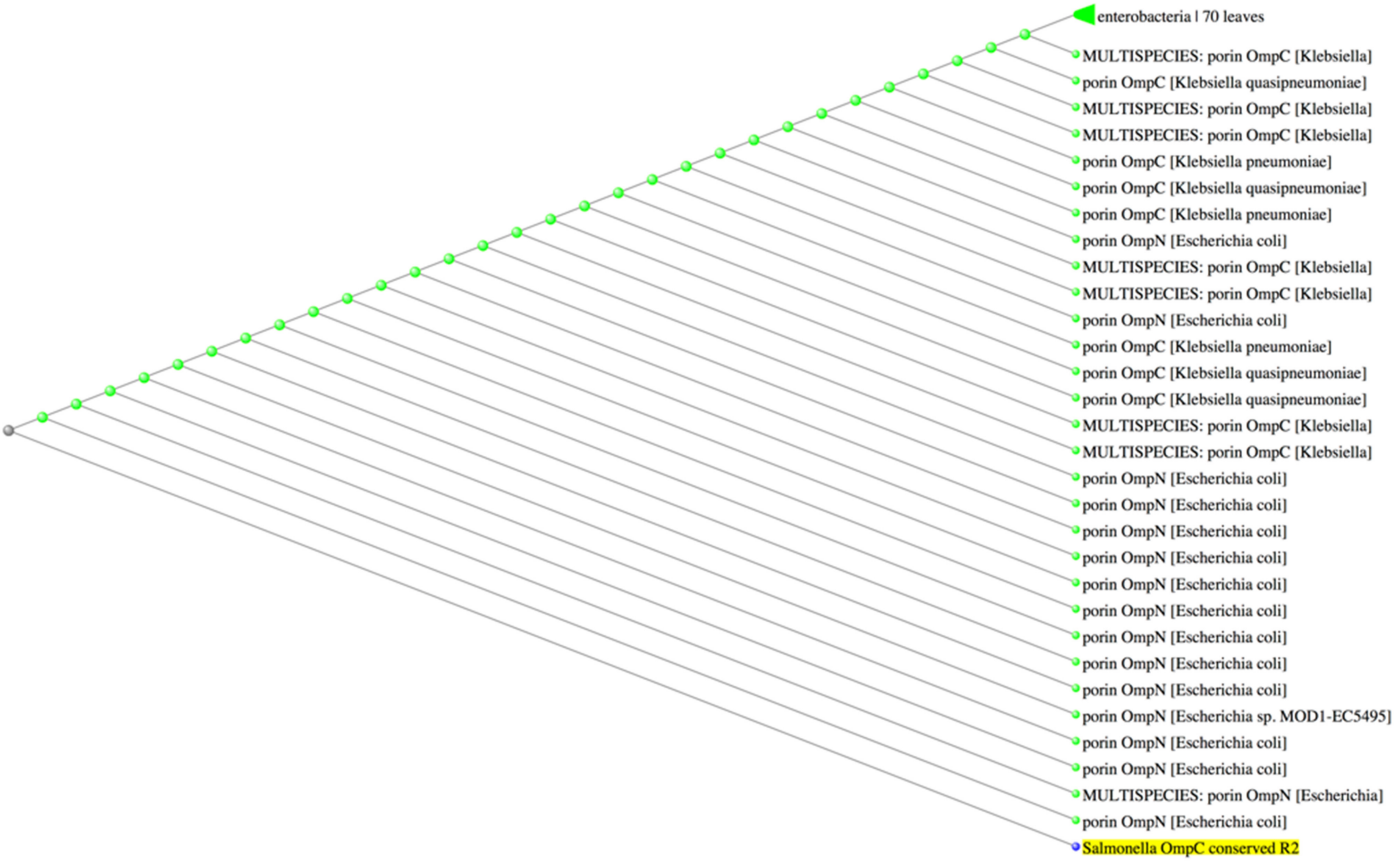
The amino acid sequence of R2 is present in *Escherichia coli* and *Klebsiella sp*. porins. Fast minimum evolution tree between the R2 sequence and the Non-Redundant (nr) GenBank database calculated by BLASTp.

**Figure 6:**
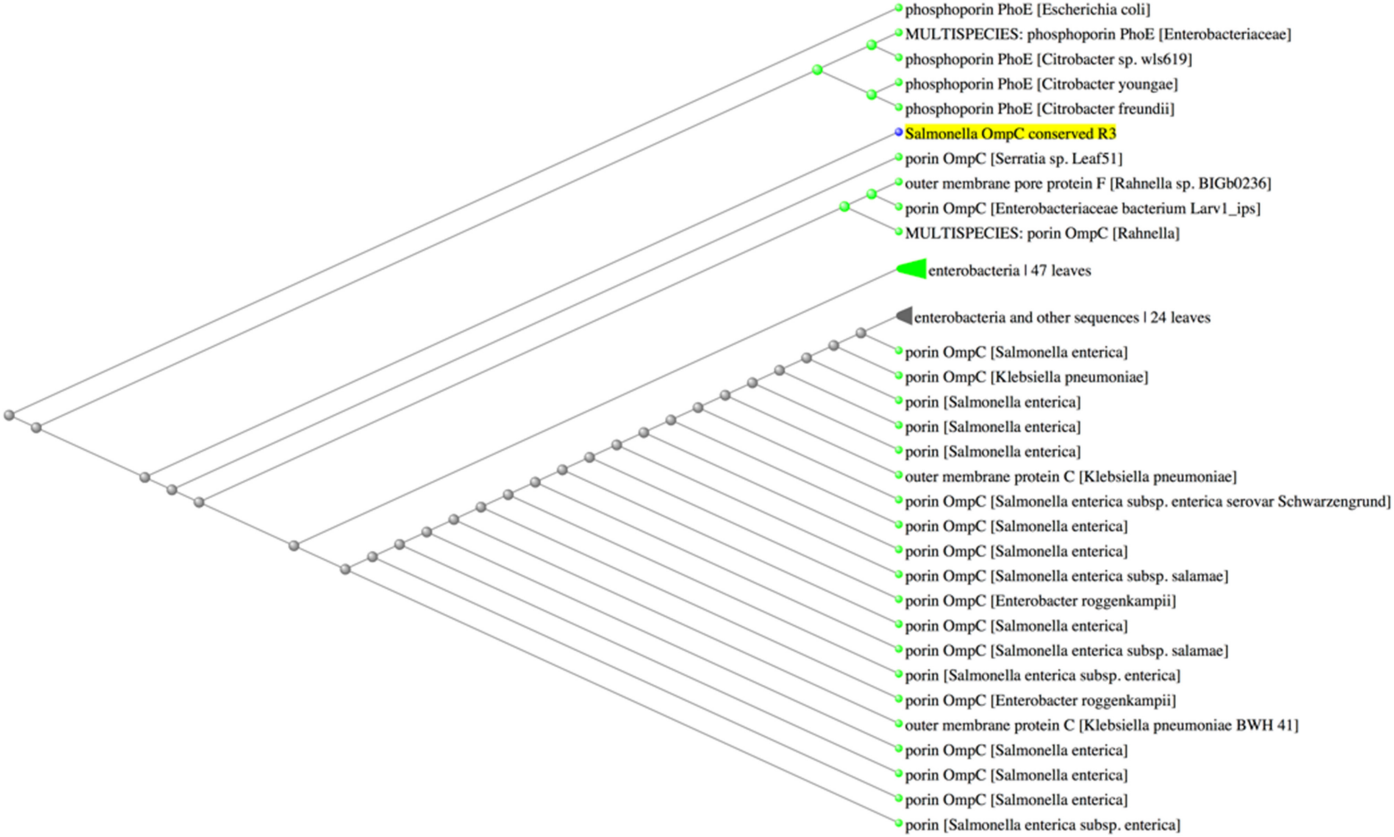
The amino acid sequence of R3 can be found in *Salmonella* and other Enterobacerial porins. Fast minimum evolution tree between the R3 sequence and the Non-Redundant (nr) GenBank database calculated by BLASTp.

Because porins from Enterobacteriaceae show high-level sequence similarity (11, 13, 15, 24), we questioned whether the conserved sequences were exclusive to *Salmonella* porin OmpC. BLASTp analysis of R1 indicated that this amino acid sequence was found among several OmpC porins from *Salmonella enterica* serovars, as well as other *Salmonella* porins, such as OmpS2 and PhoE (E-value 3 × 10^−4^, 100% identity). In addition, BLASTp results showed that the amino acid sequence from R1 (KGETQINDQLTGY) was also present in the OmpC and OmpF porins of the plant-associated genus *Pantoea* (40) (Figure 4). Conversely, the amino acid sequence of R2 (WTRLAFAGLKFA) was not exclusive to *Salmonella* serovars, since BLASTp results showed that this sequence was also found in the porins OmpC and OmpN of *Escherichia coli* and *Klebsiella sp*. (E-value 3 × 10^−3^, 100% identity) (Figure 5). Finally, the amino acid sequence of R3 (GFANKTQNFEVVAQYQFDFGLRPSQAYLSKG) was found to be present in several *Salmonella enterica* serovar OmpC porins, however it was also present in other porins, such as PhoE, OmpC, and OmpF from other Enterobacteria, such as *E. coli, Enterobacter sp., Citrobacter sp., Klebsiella sp., and Rahnella sp*. (E-value 6 × 10^−19^-4 × 10^−18^, 90.62% identity) (Figure 6). Next, based on HLA allele frequencies and reference sets with maximal population coverage, we predicted the MHC-II alleles to which the conserved R1 seqeunce could bind (Table 4).

**Table 4:** MHC-II binding prediction for HLA allele frequencies and reference sets with maximal population coverage for the conserved amino acid sequence R1 among *Salmonella* OmpC porin.

**HLA allele**	**Percentile rank**	**Adjusted rank**
HLA-DRB3^*^01:01	18	28.07
HLA-DQA1^*^01:01/DQB1^*^05:01	39.5	61.59
HLA-DQA1^*^05:01/DQB1^*^02:01	50	77.97
HLA-DRB4^*^01:01	55	85.76
HLA-DQA1^*^03:01/DQB1^*^03:02	56	87.32
HLA-DQA1^*^01:02/DQB1^*^06:02	60.5	94.34
HLA-DRB1^*^12:01	61.5	95.9
HLA-DQA1^*^04:01/DQB1^*^04:02	64.5	100.58
HLA-DRB1^*^03:01	69	107.6
HLA-DPA1^*^01/DPB1^*^04:01	69.5	108.38
HLA-DPA1^*^01:03/DPB1^*^02:01	70	109.16
HLA-DPA1^*^02:01/DPB1^*^01:01	72	112.27
HLA-DPA1^*^03:01/DPB1^*^04:02	74.5	116.17
HLA-DRB3^*^02:02	75	116.95
HLA-DRB1^*^08:02	76	118.51
HLA-DRB1^*^13:02	76	118.51
HLA-DQA1^*^05:01/DQB1^*^03:01	82	127.87
HLA-DRB1^*^04:01	85	132.55
HLA-DRB1^*^11:01	85	132.55
HLA-DPA1^*^02:01/DPB1^*^05:01	85.5	133.33
HLA-DRB1^*^04:05	86	134.1
HLA-DRB1^*^01:01	87	135.66
HLA-DRB1^*^07:01	88	137.22
HLA-DRB1^*^09:01	92	143.46
HLA-DRB1^*^15:01	92	143.46
HLA-DRB5^*^01:01	95	148.14
HLA-DPA1^*^02:01/DPB1^*^14:01	96	149.7

## Discussion

The development of novel tools for the detection of conserved sequences among vaccine candidates is particularly relevant to the discovery of shared antigenic determinants, which could be used for the rational design of broad-spectrum vaccines. In this study, we used in-house-developed software to evaluate the amino acid sequence conservation of the OmpC porin among typhoidal and NTS serovars. Although it has been reported that the porin OmpC has a high degree of homology in sequence and structure among Enterobacteriaceae porins, most of these works have focused on defining the differences among amino acid sequences between the OmpC of *Salmonella* serovars and other Enterobacteria, but have not shown the degree of conservation of OmpC among serovars (11, 13, 15, 23, 24). To our knowledge, our work is the first to determine the degree of amino acid conservation of the OmpC porin among typhoidal and NTS serovars, and is the first work to define the conserved regions among *Salmonella* serovars OmpC porin.

Previous reports have shown that the OmpC transmembrane regions are homologous in sequence and structure among Enterobacteria (13, 41). Consequently, it was expected that most of the conserved sequences among *Salmonella* OmpC would be located along the transmembrane β-sheets of the porin β-barrel, as our results show. By contrast, none of the conserved amino acid regions were located along the surface-exposed loops; furthermore, we identified a region within the OmpC porin with no conservation across clades that corresponds to the external loop L4, which has been shown to be one of the regions with more antigenic variability within the OmpC (42–44). Our results show that the *Salmonella* OmpC conserved regions are located along the transmembrane β-sheets of the porin β-barrel, one explanation for this could be that some of the amino acid sequences contained in the conserved regions R1 and R3 of *Salmonella* OmpC are located in subunit contact regions, which are highly conserved among Enterobaceriaceae porins (24, 45). Likewise, it has been reported that the arginine residue (R-95) contained in the conserved region R2 is involved in pore formation (15, 46), which could explain the conservation of this region among *Salmonella* serovars.

The evidence that the conserved sequence in R1 was exclusively found in *Salmonella* porin sequences and not in other gut-associated Enterobacteria, suggests that the immune response that this sequence would induce should be *Salmonella*-specific. Conversely, the finding that the sequences contained in regions R2 and R3 were also found in porins of other commensal and pathogenic Enterobacteria, such as *E. coli, Klebsiella sp., Enterobacter sp., Citrobacter sp., Klebsiella sp., and Rahnella sp*., suggests that the sequences of regions R2 and R3 are not exclusive of *Salmonella* porins. Our results show that the conserved OmpC sequences R1 can potentially bind to human MHC-II molecules. Similar results were found for human CD4^+^ T cell epitopes conserved between meningococcal and gonococcal *Neisseria* porins (47, 48). Furthermore, it has been reported that Enterobacteriaceae porins have crucial antigenic epitopes corresponding to regions buried within the outer membrane, which are also highly conserved among Enterobacterial species (24). Some of the amino acid residues from the OmpC conserved region R1 (GFKGETQ) have also been shown to be highly conserved among Enterobacteriaceae porins because of their location in a crucial domain involved in porin subunit interactions (24, 45). In addition, some of the amino acid sequences conserved among *Salmonella* serovars (R1 and R3), have previously been reported as antibody targets or predicted as potential B cell epitopes (11, 24, 49); however, it remains unknown whether any of the conserved sequences can also be recognized by antibodies. In addition, further studies are needed to determine the contribution of MHC-restriction responses to the immunogenicity of the conserved OmpC peptides in T cells. Previous work identified two MHC-I-restricted epitopes in *Salmonella* OmpC porin (50), and strikingly, the amino acid sequence contained in one of the CD8^+^ T cell-specific peptides contains identical or similar residues to the sequences contained in the conserved region R2 (TRVAFAGL). However, future work will need to focus on whether CD8^+^ T cells from healthy donors or convalescent patients may also recognize some of the conserved OmpC sequences.

Although this work has shed some light regarding antigen specificity of the *Salmonella* OmpC porin among typhoidal and NTS serovars, there are still several questions left unanswered. For instance, it remains to be determined the cytokine profile produced by OmpC-specific CD4^+^ T cells, as we have previously shown that vaccination of healthy volunteers with either the Ty21a vaccine or with *Salmonella* porins induces IFN-γ- and TNF-α-producing CD4^+^ T cells (20, 22). Furthermore, it remains unknown whether the conserved OmpC peptide sequences can be also recognized by T cells from convalescent patients or healthy volunteers challenged with typhoidal and NTS *Salmonella* serovars. Because our current porin-based vaccine candidate is made of a mixture of OmpC and OmpF porins, it remains to be determined the degree of conservation of the porin OmpF among typhoidal and non-typhoidal *Salmonella* by means of the same methodology.

In conclusion, our work is the first to specifically establish the degree of conservation of the porin OmpC among typhoidal and non-typhoidal *Salmonella* serovars and to define the specific amino acid sequences with the highest degree of conservation among typhoidal and NTS serovars. Furthermore, we found that one of the highly conserved OmpC amino acid sequences is exclusive for *Salmonella* and has immunogenic potential for MHC-II binding. Considering that porins are highly immunogenic and protective vaccine candidates against *Salmonella* infections, our findings may lead to a better understanding of the basis of antigen specificity of *Salmonella* porins, which could be used to design tools for monitoring the porin-specific immune response after challenge or vaccination and could have direct implications for the rational design of a broad-spectrum vaccine against *Salmonella*.

## Data Availability Statement

All datasets generated for this study are included in the article/supplementary material.

## Author Contributions

NV-P and JB performed the experiments, analyzed the results, and wrote the paper. MP-T and GA-V analyzed the results and wrote the paper. PKe wrote the in-house computer program. AK, CP-S, and CG-C analyzed results and revised the manuscript. IW-B, LS-T, RP-P, and AI analyzed the results and revised the manuscript. AR-S, PKl, and CL-M designed the study, supervised the experiments, and revised the manuscript. All authors contributed to manuscript revision, read, and approved the submitted version.

### Conflict of Interest

CL-M is listed as inventor on a patent related to the use of *Salmonella* porins as adjuvants and vaccines. The remaining authors declare that the research was conducted in the absence of any commercial or financial relationships that could be construed as a potential conflict of interest.
